# Familial Bell’s Palsy: Patient With Facial Synkinesis and Bilateral Recurrence of Facial Palsy

**DOI:** 10.7759/cureus.93488

**Published:** 2025-09-29

**Authors:** Karen Inzirillo, Roxana M Dragomir, Marc A Swerdloff

**Affiliations:** 1 Neurology, Marcus Neuroscience Institute, Boca Raton Regional Hospital, Baptist Health South Florida, Boca Raton, USA; 2 Neurology, Florida Atlantic University Charles E. Schmidt College of Medicine, Boca Raton, USA

**Keywords:** bell's palsy, facial synkinesis, familial, genetic, recurrent facial palsy

## Abstract

We present a case of Bell’s palsy involving bilateral recurrence in a family history of multiple affected individuals. Bell’s palsy, also referred to as facial palsy (FP), has been associated with infectious, ischemic, and familial cluster occurrences, yet its etiology remains largely unclear. Familial clustering, also referred to as familial Bell’s palsy, may indicate an underlying anatomical abnormality of the facial canal or an immune-mediated mechanism contributing to a hereditary predisposition. However, this potential link remains understudied. Regardless of the underlying cause, the diagnosis and management of FP follow standardized protocols. Our patient contributes to the growing body of evidence suggesting that familial FP may be a more prevalent cause than previously recognized. Therefore, obtaining a detailed family history is essential when evaluating cases of FP to identify any hereditary component. Familial FP tends to have a higher likelihood of recurrence and may lead to synkinesis.

## Introduction

Facial palsy (FP) is characterized by spontaneous unilateral or bilateral lower motor neuron paralysis of the seventh cranial nerve. The facial nerve controls the muscles of facial expression and relays taste fibers from the anterior two-thirds of the tongue. FP has an annual incidence of 13-52 per 100,000 people globally [1-4]. The exact cause is uncertain [3,5-7] but may arise from inflammation or compression of the facial nerve along its course in the facial canal. Risk factors include pregnancy, infection, small vessel disease (from diabetes or hypertension), or a genetic predisposition [3,4,6-11]. The prevailing theory for familial Bell's palsy suggests that it is due to an inherited anatomical abnormality in the facial canal [2,4,5,7,9]. Another genetic theory (from the 1970s to the 1990s) suggests a heritable susceptibility mediated by human leukocyte antigen (HLA)-linked immune response genes [2,4,11,12]. To date, no specific gene has been identified as a definitive cause of Bell’s palsy [5]. Familial FP prevalence reported ranges from 4% to 14% or 2.4% to 28.6%, depending on the study [2,7,8,11].

A quarter of cases have fiber misdirection during FP recovery, resulting in facial synkinesis [5]. There is an inability to isolate individual facial regions due to co-contraction of all the previously weak facial muscles. Thus, attempted eye closure will result in wrinkling of the chin, lip movement, and a twitch of the anterior neck muscles. This broad activation is frequently misinterpreted by the patient as a recurrence of the illness and can be distressing. 

Recurrent FP occurs in 2.6-15.3% of cases [3,4,11,12] and is greater in familial cases [2,4,6,7,10], which increases the risk of facial synkinesis (55% of cases) [13]. The treatment of familial FP is identical to nonfamilial cases. It includes corticosteroids, antivirals, eye care, and physical therapy. Botulinum toxin injections relax overactive muscles and reduce involuntary muscle contractions in facial synkinesis [3].

## Case presentation

**Figure 1:**
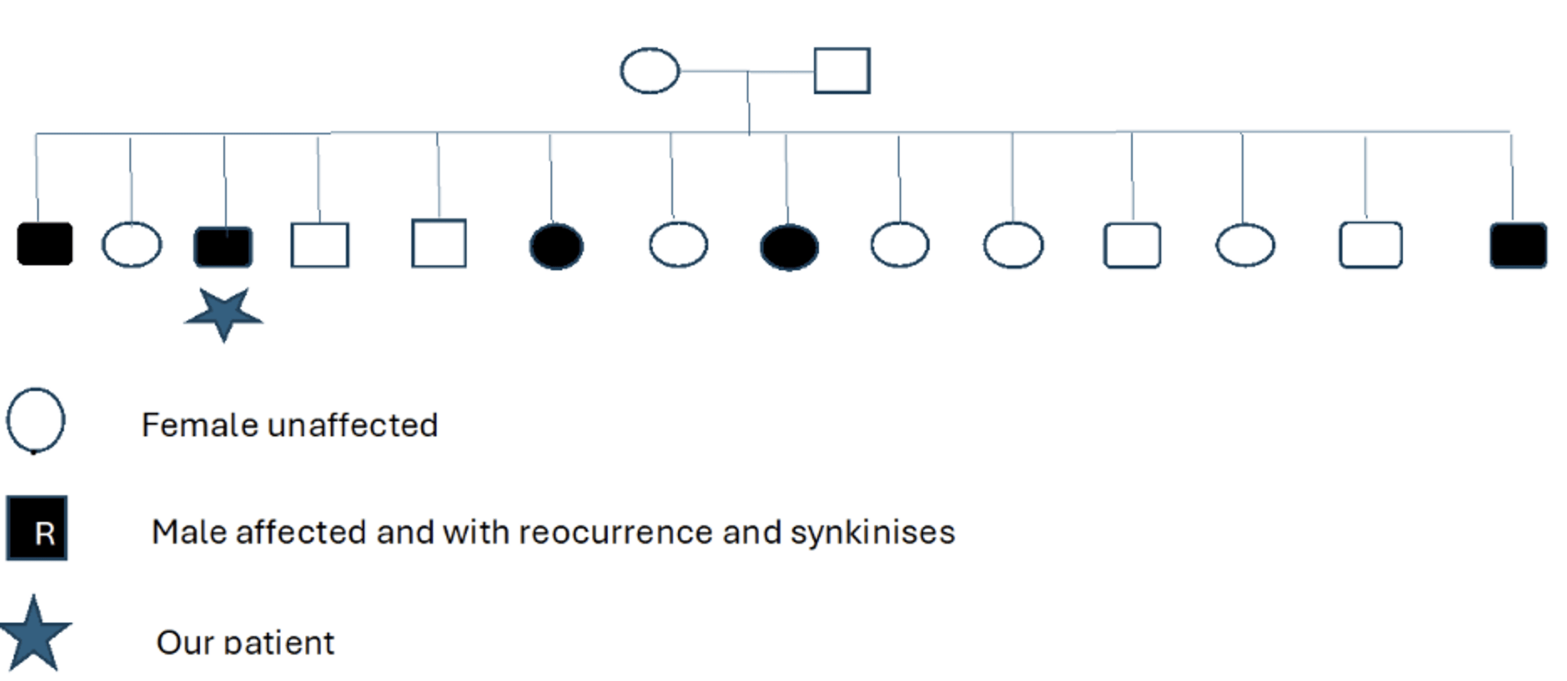
Family tree of our patient with five affected family members with Bell’s palsy across one generation This is consistent with either an autosomal recessive inheritance pattern or autosomal dominant with incomplete penetrance (assuming low penetrance in the parents)

A 53-year-old male patient with a history of hypertension presented to the emergency room with left-sided facial numbness, flattening of the left nasolabial fold and difficulty closing his left eye. A magnetic resonance imaging of the brain was negative. Blood laboratories, including complete blood count (CBC), comprehensive metabolic panel (CMP), and sed rate results, were within normal limits. He was diagnosed with FP and treated with antiviral (acyclovir 400 mg five times daily for 10 days) and oral corticosteroid (prednisone 50 mg per day for five days, followed by a five-day taper). Upon recovery, he developed left facial synkinesis resulting in a narrowed left palpebral fissure and synkinesis manifested as co-contraction of eye closure, left platysma, and left frontalis. At age 64, he had contralateral FP heralded by pain behind his right ear, closely followed by right facial weakness with incomplete right eye closure. He noted right-sided hyperacusis and dysgeusia. He was treated again with antiviral medications and steroids acutely. Right facial strength improved, joining his previous left facial synkinesis. Five months later, right facial synkinesis developed with complaints of right cheek pain during mastication and annoying curling of his right lower lip while speaking or chewing (Video 1). Facial sensation remained unaffected. He reported that five of his fourteen siblings had experienced FP, including three males and two females. Two of the affected males, both in their 50s and 60s, had recurrent episodes accompanied by synkinesis. The two females, in their 40s, and one younger male sibling experienced FP without recurrence or synkinesis. The medical histories and treatment details of these family members are currently unknown (Figure 1). 

**Video 1:** Facial features showing established left synkinesis and newly developed right facial synkinesis

## Discussion

Bell’s FP is characterized by spontaneous unilateral or bilateral lower motor neuron paralysis of the seventh cranial nerve. This nerve controls the muscles of facial expression and relays taste fibers from the anterior two-thirds of the tongue. 

FP presents with an asymmetric smile (drooling when severe), variable degrees of inability to close the eye, dysgeusia, decreased tearing, aural hypersensitivity, and ipsilateral retroauricular or mandibular pain. The exact cause often remains unknown. In most instances, the condition resolves in 3-6 months with no or minimal residua [3,4,12]. Residual eyelid weakness may result in corneal scarring with visual loss [3,5,12]. 

The etiology, pathogenesis, treatment, and prognosis of recurrent FP have been less studied than sporadic [13]. Autosomal dominant inheritance with high penetrance has been described in a three-generation family [4,7-10]. Low penetrance in families affecting just one generation has been reported [2,4,10,11] in addition to our patient case. 

In some cases, FP may recur on the same or opposite side of the face, sometimes leaving residual deficits. This can be distressing for patients, causing physical, psychological, and physiological challenges [13]. Knowing the patient's family and personal history of previous episodes of FP increases suspicion for inherited susceptibility. If there is a familial component, the risk of recurrence and the development of facial synkinesis increases. The treatment approach for FP is the same regardless of whether it is familial or nonfamilial. 

## Conclusions

FP, particularly Bell’s palsy, is a neurologic condition with a multifactorial etiology and unpredictable prognosis. While most cases resolve spontaneously, recurrent and familial forms present greater clinical challenges due to the increased likelihood of residual deficits such as facial synkinesis. Genetic predisposition, potentially involving anatomical variations or immune-mediated mechanisms, may contribute to familial cases, although no definitive gene has been identified. Recognizing a patient’s personal and family history is essential for anticipating possible recurrence. Our patient illustrates bilateral recurrence of FP with synkinesis. His positive family history of FP in five of his fourteen siblings, including two with recurrent synkinesis, supports a possible hereditary component. Continued research into the genetic and immunologic basis of FP is needed to enhance diagnostic accuracy and therapeutic strategies.
